# Cost‐Effectiveness of Decompressive Craniectomy in Severe Traumatic Brain Injury Patients: A Systematic Review of Economic Studies

**DOI:** 10.1002/brb3.71748

**Published:** 2026-09-06

**Authors:** William Andrés Florez‐Perdomo, Juan Sebastián Reyes Bello, Luis Rafael Moscote‐Salazar, Vishal Chavda, Kashif Qureshi, Murtaja Satea, Claudia Restrepo, Amit Agrawal, Daniel Ant Encarnacion Santos, Mukhammadjon Norov, Norov Abdurakhmon, Bipin Chaurasia

**Affiliations:** ^1^ Department of Research Colombian Clinical Research Group in Neurocritical Care Bogotá Colombia; ^2^ Department of Neurology, Neurosurgery and NeuroGenetics, Department of Clinical and Translational Medicine ChromoMed Institute Santo Domingo Dominican Republic; ^3^ Department of Neuroscience and Neurosurgical Surgery Neurotraumatology Clinic New Mexico Dominican Republic; ^4^ Department of Neurosurgery Yale School of Medicine New Haven Connecticut USA; ^5^ Department of Neurosurgery, College of Medicine University of Warith Al‐Anbiyaa Karbala Governorate Iraq; ^6^ Department of Neurosurgery All India Institute of Medical Sciences, Saket Nagar Bhopal Madhya Pradesh India; ^7^ Department of Neurosurgery Russian People's Friendship University, Named after Patrice Lumumba Moscow Russia; ^8^ Department of Spinal Neurosurgery Republican Specialized Scientific and Practical Medical Center of Neurosurgery Tashkent Uzbekistan; ^9^ Department of Neurosurgery Neurosurgery Clinic Birgunj Nepal

**Keywords:** cost, cost analysis, cost‐effectiveness, decompressive craniectomy, intracranial pressure, traumatic brain injury

## Abstract

**Objective:**

This systematic review aimed to assess the cost‐effectiveness of decompressive craniectomy (DC) in severe traumatic brain injury (TBI) patients by analyzing relevant economic studies.

**Methods:**

The authors conducted a comprehensive search across multiple databases and included economic evaluation studies, clinical trials, observational studies, or modeling studies that focused on patients who underwent DC for severe TBI. The quality of included studies was assessed using the Drummond checklist, and potential publication bias was examined. Data on total hospital costs, quality‐adjusted life years (QALY), cost per QALY, and patient demographics were extracted and analyzed.

**Results:**

The included studies were of moderate to high quality. Total hospital costs increased with a higher likelihood of unfavorable outcomes. A cost–utility analysis demonstrated that DC led to a higher expected QALY gain compared to medical management. At 12 months, though, DC was the costlier option. In the Finnish cohort, the estimated cost of continued care ran to several times that of the initial admission, which raised the all‐in cost per QALY well above the figure for neurosurgical treatment alone. Cost‐effectiveness varied with the severity of TBI.

**Conclusions:**

DC may be cost‐effective in the management of severe TBI in selected patients, particularly those at lower risk of an unfavorable outcome, although the available evidence is limited and heterogeneous. That value falls as injury severity rises, and at the extreme end of severity the procedure is no longer cost‐effective. Decisions about DC in severe TBI therefore turn on both the expected clinical benefit and the cost, not on either alone. Longer follow‐up would help, since much of both the cost and the recovery in these patients accrues years after surgery.

## Graphical abstract

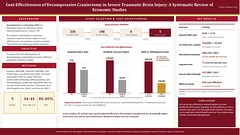

## 1 INTRODUCTION

In patients with traumatic brain injury (TBI), particularly with severe injuries, the management of raised intracranial pressure (ICP) is a complex multistep process and requires maintaining the ICP below 25 mmHg and thus maintaining the cerebral perfusion pressure (CPP) between 60 and 70 mmHg (Dash and Chavali 2018). In initial phases a raised ICP can be managed by head elevation, sedation, analgesia, temperature, and fluid control (Dash and Chavali 2018), which can be escalated further if the patients do not respond to initial management. In refractory cases or where there is a significant rise in ICP, decompressive craniectomy (DC) plays a role in preventing further neurological deterioration (Vella et al. 2017; Fatima et al. 2019). Although the results are variable across the studies (Cooper et al. 2011; Olivecrona et al. 2007; Soustiel et al. 2010; Lemcke et al. 2010), however, studies have shown (Hutchinson et al. 2016) that DC lowers mortality in refractory intracranial hypertension. With respect to cost‐effectiveness, management strategies in clinical medicine should be evaluated with the same basic principles (Owens et al. 2011) so clinical evidence can be generated to formulate policies to optimally allocate resources particularly when these are limited (Bochner et al. 1994; Cookson and Dolan 2000; Daniels et al. 2009; Fuchs 2010; Relman 1990; Alexander et al. 2004). Although DC is globally used as a lifesaving procedure in the management of severe TBI, only a few studies have reported its cost‐effectiveness, and overall estimates remain uncertain (Ho et al. 2011). In the present article, we systematically review the literature and examine the pattern of reporting on the cost‐effectiveness of DC in the management of patients with severe TBI.

## 2 METHODS

### 2.1 Search

The following databases were searched for systematic reviews: EMBASE (Ovid), MEDLINE (Ovid), the Cochrane Central Register of Controlled Trials (CENTRAL), and LILACS, all searched to July 2026 in addition to the reference list of included studies and other relevant data in addition to potentially eligible studies. No date or language restrictions were applied. Terms used: (“decompressive craniectomy” OR “bifrontal craniectomy” OR “temporal Craniectomy”) AND (“traumatic brain injury” OR “cerebral trauma” OR “head trauma”) AND (“cost‐effectiveness” OR “cost‐benefits” OR “cost‐utility” OR “cost”) (Supplementary File S1).

### 2.2 Selection Criteria

#### 2.2.1 Inclusion Criteria

1. Economic evaluation study design that was either prospective or retrospective alongside a clinical trial, observational studies, or based on modeling.
2. Patients with severe TBI treated with DC
3. Included cost‐effectiveness, cost–utility, or cost‐benefit analysis.

#### 2.2.2 Exclusion Criteria

1. Non‐TBI conditions such as stroke
2. Patients without DC

### 2.3 Quality Assessment of Included Studies

The 10‐item Drummond checklist (Drummond et al. 2015) was used to assess the methodological quality of the economic evaluation studies. If the study met any of these 10 items, it would be considered as “Yes,” otherwise “No,” or “Cannot tell.” The Drummond checklist provides a global assessment of the quality of evidence. The quality of the included studies was assessed based on this checklist as “High” 8–10 answered as yes in the checklist, “moderate” 5–7 items yes: “Low” 4 or less items in the checklist (Table 1).

**TABLE 1:** Quality assessment with the 10‐item Drummond checklist (Drummond et al. 2015).

Checklist
Was a well‐defined question posed in answerable form? Yes/No/NR^a^ (not reported)
Was a comprehensive description of the competing alternatives given (i.e., can you tell who did what to whom, where, and how often)? Yes/No/NR^a^
Was the effectiveness of the program or services established? Yes/No/NR^a^
Were all the important and relevant costs and consequences for each alternative identified? Yes/No/NR^a^
Were costs and consequences measured accurately in appropriate physical units (e.g. hours of nursing time, number of physician visits, lost work‐days, gained life years)? Yes/No/NR^a^
Were the cost and consequences valued credibly? Yes/No/NR^a^
Were costs and consequences adjusted for differential timing? Yes/No/NR^a^
Was an incremental analysis of costs and consequences of alternatives performed? Yes/No/NR^a^
Was allowance made for uncertainty in the estimates of costs and consequences? Yes/No/NR^a^
Did the presentation and discussion of study results include all issues of concern to users? Yes/No/NR^a^

^a^ NR: not reported.

The Drummond checklist evaluates the methodological quality of economic evaluations rather than the risk of bias; design‐specific risk‐of‐bias tools such as ROBINS‐I for observational studies and RoB 2 for randomized trials were not applied, which we acknowledge as a limitation. The high, moderate, and low quality categories used here were defined by the authors for the purpose of this review and are not part of the original Drummond framework. These thresholds divide the ten checklist items into approximate thirds, so that studies meeting most of the items (8 to 10) were rated high quality, those meeting roughly half to two thirds (5 to 7) moderate quality, and those meeting fewer than half (4 or fewer) low quality.

Given the small number of included studies, a formal assessment of publication bias, such as funnel‐plot asymmetry or Egger's test, was not performed, because these methods are unreliable when fewer than ten studies are available.

### 2.4 Extraction, Management, and Statistical Analysis of Data

Individually and separately, the following data were extracted: total hospital cost in American dollars (if the study was expressed in another currency, approximate conversion was made into dollar values) included all cost of trauma patient's health attention (presurgical, craniectomy, and postsurgical care), QALY (quality‐adjusted life year), cost for QALY (US$ per QALY) (included craniectomy, cost of rehabilitation and another cost); authors were contacted for missing and not reported data. Disagreements were resolved by consensus. Because the included studies differed substantially in design, comparator, currency, price year, and unit of analysis, a pooled quantitative synthesis was not appropriate. The findings were therefore summarized using a structured narrative synthesis, with costs and cost‐effectiveness reported in each study's original currency and, where converted by the original authors, in US dollars at the exchange rate of the study year without inflation adjustment.

## 3 RESULTS

### 3.1 Study Selection

**FIGURE 1:**
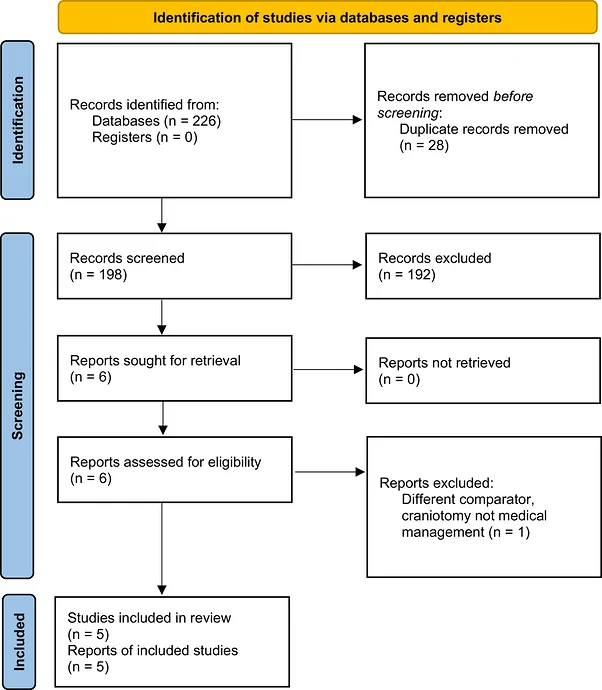
Process of study selection—flow chart of search strategy and inclusion and exclusion criteria.

Following a systematic search according to the predefined strategy, a comprehensive search updated to July 2026 identified 226 records across the databases (Embase and MEDLINE, Cochrane CENTRAL, and LILACS). After 28 duplicate records were removed, 198 records were screened by title and abstract, and 192 were excluded as clinical or outcome studies, cranioplasty or bone‐flap cost analyses, reviews, protocols, or commentaries that were not economic evaluations of DC in TBI. Six reports were assessed at full text, and one was excluded because its comparator was craniotomy rather than medical management. Five research papers were included for the narrative synthesis (Ho et al. 2011; Alali et al. 2014; Badke et al. 2018; Behranwala et al. 2021; Malmivaara et al. 2011) focused on the cost‐effectiveness of DC in severe TBI cases (Figure 1). The characteristics of the included studies are shown in Table 2.

**TABLE 2:** Characteristics of included studies.

Study	City and country	Clinical evidence (source, results)	Methods	Cost‐effectiveness
Malmivaara et al. (2011)	Helsinki, Finland	Patients (*n* = 54)	Retrospective observational study	Quality‐adjusted life years (QALY) Euro (€) per QALY Total hospital cost (€)
Ho et al. (2011)	Perth, Western Australia, Australia	Patients (*n* = 168)	Retrospective observational study	Quality‐adjusted life years (QALY) American dollars (US $) per QALY Total hospital cost (US $)
Alali et al. (2014)	Toronto, Ontario, Canada	Data base of University of Toronto and governmental Statistic	Markov models projection 1000 patients	Quality‐adjusted life years (QALY) American dollars (US $) per QALY Total hospital cost (US $)
Badke et al. (2018)	São Paulo, Brazil	Patients (*n* = 32)	Retrospective observational study	Total Hospital cost in Brazilian real (R$)
Behranwala et al. (2021)	London, England, United Kingdom	Previous Randomized clinical trials	Markov models projection 1000 patients	Quality‐adjusted life years (QALY) Pound sterling (£) per QALY Total hospital cost (£)

### 3.2 Quality Evaluation of Economic Studies

According to the 10‐item Drummond checklist (Drummond et al. 2015), the methodological quality of the included studies was moderate to high, with two studies rated high quality (Ho et al. 2011; Alali et al. 2014) and three rated moderate quality (Badke et al. 2018; Behranwala et al. 2021; Malmivaara et al. 2011) (Table 3).

**TABLE 3:** Assessment of quality in the included study: Drummond checklist for quality evaluation of economic studies.

Studies	1	2	3	4	5	6	7	8	9	10	Total items	Quality judgment
Malmivaara et al. (2011)	Yes	Yes	Yes	Yes	Yes	NR	Yes	Yes	No	NR^a^	7/10	**Moderate**
Ho et al. (2011)	Yes	Yes	Yes	No	Yes	Yes	Yes	Yes	No	Yes	8/10	**High**
Alali et al. (2014)	Yes	NR	Yes	Yes	Yes	Yes	Yes	Yes	Yes	Yes	9/10	**High**
Badke et al. (2018)	Yes	Yes	Yes	NR	Yes	No	Yes	No	Yes	Yes	7/10	**Moderate**
Behranwala et al. (2021)	Yes	No	Yes	Yes	NR	Yes	Yes	Yes	Yes	No	7/10	**Moderate**

^a^ NR: not reported.

### 3.3 Patient Demographics

Patients involved in the studies were predominantly young (with a mean age ranging from 34 to 44 years) and mostly male (with percentages ranging from 84.4% to 85%).

A significant proportion of patients had severe head injuries and were in poor neurological condition, often indicated by a low Glasgow Coma Scale (GCS) score (< 8).

### 3.4 Total Hospital Costs

It was observed that total hospital costs increased when there was a higher likelihood of an unfavorable outcome (Table 4). Patients with a greater predicted risk of unfavorable outcomes and those with more severe and disabling TBI incurred significantly higher total hospital costs (Table 4).

**TABLE 4:** Summary of the economic findings of the included studies (descriptive synthesis).

Study	Country/design	Comparator	Costs (as reported in the original study)	Cost per QALY/ICER (as reported)	Authors' conclusion
Malmivaara et al. (2011)	Finland/retrospective, single‐arm	None (single‐arm)	Neurosurgical treatment 2,101,000 EUR (all 54 patients); total estimated continued‐treatment cost 15.7 million EUR (about seven times the primary‐treatment period)	2400 EUR per QALY (neurosurgical treatment); 17,900 EUR per QALY (all costs, including rehabilitation and future care)	Cost per QALY gained was acceptable (below the 50,000 EUR threshold)
Ho et al. (2011)	Australia/retrospective	Withholding the procedure (implicit)	Total hospital costs rose with severity and peaked at about 70% to 80% predicted risk of an unfavorable outcome	US$140,000 per QALY at predicted risk 80% or below, rising to US$682,000 per QALY at risk above 80% (usual acceptable limit US$100,000)	Not cost‐effective for patients with extremely severe TBI
Alali et al. (2014)	United States/Markov microsimulation (base age 25)	Barbiturate coma	Discounted lifetime cost US$132,564 (craniectomy) vs. US$117,780 (barbiturate coma); incremental US$14,784	ICER US$9,565 per QALY (1.5 QALY gained); rising to US$197,906 per QALY at age 85	Cost‐effective vs. barbiturate coma at a US$50,000 per QALY threshold
Badke et al. (2018)	Brazil/retrospective, single‐arm	None (single‐arm)	Total R$2116960.22 (US$661550.06); mean US$20673.44 per patient (range US$2151.20 to 96599.94)	Not reported	Expensive procedure associated with high morbidity and mortality
Behranwala et al. (2021)	United Kingdom/Markov model, 1‐year horizon, NHS perspective	Maximal medical management	Expected cost 16034.36 GBP (craniectomy) vs. 6407.00 GBP (medical management); difference 9627.36 GBP	ICER 96155.67 GBP per QALY (0.41 vs. 0.31 QALY); NICE threshold 30,000 GBP per QALY	Not cost‐effective as an initial treatment

### 3.5 Cost–Utility Analysis (CUA)

In one study (Behranwala et al. 2021), a cost–utility analysis compared DC with medical management for cases of refractory traumatic intracranial hypertension. Patients undergoing DC showed a higher expected quality‐adjusted life year (QALY) gain (0.41) compared to medical management (0.31) (Table 4). At 12 months, the cost of DC was notably higher than that of medical management.

### 3.6 Long‐Term Costs

In a separate study (Malmivaara et al. 2011), the total estimated cost of continued treatment for patients who underwent DC was significantly higher than the initial treatment period cost (Table 4). The cost per QALY in that study was correspondingly high, driven largely by the extended cost of continued care (Table 4).

### 3.7 Cost‐Effectiveness and Severity of TBI

Cost‐effectiveness was tightly coupled to injury severity, with more severe TBI yielding a less favorable cost per QALY (Table 4). For patients with extremely severe TBI, DC was found to be less cost‐effective (Ho et al. 2011).

## 4 DISCUSSION

In a study, the authors analyzed clinical data of 168 patients who underwent DC in two neurotrauma centers for severe neurotrauma (Ho et al. 2011). Majority of the patients in the study were young (mean age 34 years) male (85%) (Ho et al. 2011). In addition to management of underlying injuries, the patients were also treated for associated postoperative complications, that is, infected bone flap, seizures, and management of post‐traumatic hydrocephalus (Ho et al. 2011). The average total hospital cost increased when there was a prediction of an unfavorable outcome (Ho et al. 2011). Patients who had an increased predicted risk of an unfavorable outcome >80%; and as the severity and disability associated with TBI increased the total hospital costs were much higher. Even if the patients survived and had good recovery, the costs per life‐year gained and QALY remained very high (Ho et al. 2011)

Behranwala et al. (2021) carried out a cost–utility analysis (CUA) in patients who underwent DC and compared it versus medical management in the management of refractory traumatic intracranial hypertension. Patients who underwent DC had an expected QALY gain of 0.41, which was more than that of medical management (0.31). The Markov model demonstrated that both the expected cost and benefit of DC as initial treatment for TBI exceed that of initial medical management (Behranwala et al. 2021). At 12 months, the expected cost of the DC was £16034.36 whereas the cost of medical management was £6407.00, so decompressive craniectomy exceeded medical management by £9627.36. The incremental cost‐effectiveness ratio was £96155.67 per QALY, which exceeds the National Institute for Health and Care Excellence threshold of £30,000 per QALY; the authors concluded that DC is not cost‐effective as an initial treatment for refractory intracranial hypertension (Behranwala et al. 2021).

Alali et al. (2014) performed a cost–utility analysis comparing DC with barbiturate coma for refractory intracranial hypertension, using a Markov microsimulation model with a lifelong time horizon. In the base case (mean age 25 years), DC was associated with a discounted lifetime cost of US$132,564 compared with US$117,780 for barbiturate coma, an incremental cost of US$14,784 and an average gain of 1.5 QALYs, giving an incremental cost‐effectiveness ratio of US$9565 per QALY. At a willingness‐to‐pay threshold of US$50,000 per QALY, DC was the more cost‐effective option in the majority of simulations, and the authors concluded that DC was cost‐effective relative to barbiturate coma. Cost‐effectiveness decreased with increasing age, with the incremental cost‐effectiveness ratio rising to US$197,906 per QALY at a mean age of 85 years.

In a series of 5223 surgeries, 133 DCs were performed, and randomly 32 patients who underwent DC for TBI were selected (Badke et al. 2018). The male gender predominated (84.4%), the average age was 44 years and most of them (65.6%) had severe head injury. Length of stay and injury severity both tracked with total cost in this cohort; a 60‐day admission, for instance, carried an estimated cost of $28886.05 (Badke et al. 2018). Of the 10 survivors, five had a good outcome (Glasgow Outcome Scale score of 4 or 5). For the whole cohort, the total cost was US$661550.06, and the mean patient cost was US$20673.44 (SD ± US$22255.37 and range US$2151.20 to 96599.94) (Badke et al. 2018). When the minimum Brazilian wage was considered, that is, US$292.81 per month this amount was quite high for a person (Badke et al. 2018).

In a series of 54 patients who underwent DCs majority of the patients were young males (mean age 37 years (SD ± 14 years, 44 were male) and prior to injury were in good health (Malmivaara et al. 2011). At the time in addition to a spectrum of intracranial injuries all had intractable cerebral edema and were in poor neurological condition (45 of 54 had a Glasgow coma scale score < 8) (Malmivaara et al. 2011). In many of the patients, the decision to perform DC was based on the status of the pupillary reaction. Overall mortality in the series was 41% (Malmivaara et al. 2011). Estimated total costs included initial management and future costs as well and the total estimated cost of continued treatment was 15.7 million €, which is seven times the costs of the primary treatment period. The estimated cost was €17,900 per QALY (Malmivaara et al. 2011). Twenty‐six percent of patients had early complications including post‐operative hematomas, hydrocephalus (that was treated with ventriculostomy), meningitis, and epilepsy in survivors that required additional medications. Patients who did not develop complications were likely to have good outcomes (47% vs. 25%) (Malmivaara et al. 2011).

### 4.1 Cost‐Effectiveness

The severity of TBI had an important effect on the cost‐effectiveness of DC. As a lifesaving procedure, DC was not cost‐effective for patients with extremely severe TBI (Ho et al. 2011; Drummond et al. 2015; Alali et al. 2014; Badke et al. 2018; Behranwala et al. 2021; Malmivaara et al. 2011; Raja and Chaurasia 2024; Bozkurt et al. 2022).

It should be emphasized that higher healthcare costs do not necessarily indicate poorer cost‐effectiveness, since cost‐effectiveness reflects the balance between costs and clinical benefit rather than cost alone. The available evidence is limited and derives from heterogeneous healthcare systems, and these findings should therefore be interpreted with caution. More recent analyses, including a United Kingdom cost‐effectiveness study of craniotomy versus DC for traumatic acute subdural hematoma (Pyne et al. 2024) and a contemporary appraisal of DC in severe TBI (Raoufi et al. 2025), provide additional context but do not directly replicate the objectives of the present review.

## 5. Limitations

The findings of this economic evaluation should be considered in the context of several limitations. Firstly, the utility values associated with each GOS‐E score were taken from a study by Kosty and colleagues, which was subject to key biases. Despite adopting the reliable standard gamble approach to ascertain quality of life, the utility estimates are limited by the non‐representative population sample used to elicit preferences, unevenly distributed in age and education level. Variables such as education level and age can affect individual risk aversion with differing directions of causality; this was unaccounted for in the utility scores.

Furthermore, another limitation of the trial data used for this economic evaluation is that 37.2% of patients in the medical group underwent DC. This situation may have understated the observed treatment effect of DC as outcomes were reported in the intention‐to‐treat population. Therefore, the average QALY gained by patients randomized to the medical group may have been overstated, which could have inflated the ICER.

An additional limitation of this analysis lies in the 1‐year time horizon considered for post‐TBI outcomes, as this was the time reported in Hutchinson et al.’s study. Future cost–utility analyses could explore randomized controlled trials reporting on a longer time frame of outcomes to account for further accumulated costs.

Several further limitations should be acknowledged. The methodological quality of the included studies was appraised with the Drummond checklist, which evaluates the quality of economic evaluations rather than the risk of bias; no design‐specific risk‐of‐bias instrument (ROBINS‐I for observational studies or RoB 2 for randomized trials) was applied, and this is an important limitation, because the resulting quality ratings should not be interpreted as risk‐of‐bias assessments. Only five studies were available for synthesis, and they differed substantially in design, comparator, healthcare system, currency, price year, and time horizon, which limits their comparability. Costs were converted to US dollars without adjustment for inflation or a common price year, so values collected between 2010 and 2021 are not directly comparable. The absence of inflation adjustment and standardization to a common price year is therefore an important limitation of this review. Because fewer than ten studies were included, formal assessment of publication bias through funnel‐plot asymmetry and Egger's test is underpowered and should be regarded as non‐informative. These constraints limit the strength of any pooled estimate and support a cautious, primarily descriptive interpretation of the evidence. In addition, this review was not prospectively registered, and the literature search was updated to July 2026. One further economic evaluation identified in this update (Pyne et al. 2024) compared DC with craniotomy rather than with medical management and was therefore not added to the primary synthesis.

## 5 Conclusions

In selected patients, particularly those at lower predicted risk of a poor outcome, DC can be cost‐effective. Whether it is depending heavily on the comparator, the healthcare system, the patient's prognosis, and the willingness‐to‐pay threshold, and the evidence base remains small and mixed. Costs were highest in the most severely injured patients, namely those with a poor predicted prognosis, more severe injury, and greater residual disability. DC yielded more quality‐adjusted life years than medical management, but at markedly higher 12‐month cost, so the long‐term financial burden cannot be ignored.

Outcomes measured over a longer‐time horizon may provide greater insight into the cost‐effectiveness of DC as recovery from TBI can occur over a period of several years.

## Author Contributions

All authors (WAFP, JSRB, LRMS, VC, CR, AA, DES, and BC) have contributed equally. KQ, MS, MN, and AN helped in writing, reviewing, and final editing.

## Funding

The authors have nothing to report.

## Conflicts of Interest

The authors declare no conflicts of interest.

## Supporting information




**Supplementary Material**: brb371748‐sup‐0001‐S1.docx

## Data Availability

The authors have nothing to report.
