# Supplementary material for: Cost‐Effectiveness of Decompressive Craniectomy in Severe Traumatic Brain Injury Patients: A Systematic Review of Economic Studies
Source: Brain Behav. 2026 Sep 6;16(9):e71748. doi: 10.1002/brb3.71748 (PMC13547744; doi:10.1002/brb3.71748)
Supplement: Supplementary file 1 [file BRB3-16-e71748-s001.docx]

# **Supplementary Material S1. Search Strategies**

Systematic review of the cost-effectiveness of decompressive craniectomy in severe traumatic brain injury. Searches were run from database inception to July 2026, with no date or language restrictions. The strategy combined three concept blocks (decompressive craniectomy; traumatic brain injury; cost or economic evaluation) with the Boolean operator AND.

## **1. MEDLINE (Ovid), 1946 to July 2026**

1. Decompressive Craniectomy/

2. (decompressive adj2 craniectom*).tw,kf.

3. (bifrontal craniectom* or temporal craniectom* or hemicraniectom*).tw,kf.

4. 1 or 2 or 3

5. Brain Injuries, Traumatic/ or Craniocerebral Trauma/

6. (traumatic brain injur* or head injur* or head trauma or cerebral trauma or TBI).tw,kf.

7. 5 or 6

8. exp "Costs and Cost Analysis"/

9. (cost* or cost-effective* or cost-utility or cost-benefit or economic evaluation or QALY or quality-adjusted).tw,kf.

10. 8 or 9

11. 4 and 7 and 10

## **2. PubMed, to July 2026**

("Decompressive Craniectomy"[Mesh] OR "decompressive craniectomy"[tiab] OR

"bifrontal craniectomy"[tiab] OR "temporal craniectomy"[tiab] OR hemicraniectomy[tiab])

AND ("Brain Injuries, Traumatic"[Mesh] OR "Craniocerebral Trauma"[Mesh] OR

"traumatic brain injury"[tiab] OR "head injury"[tiab] OR "head trauma"[tiab] OR

"cerebral trauma"[tiab] OR TBI[tiab])

AND ("Costs and Cost Analysis"[Mesh] OR cost*[tiab] OR "cost-effectiveness"[tiab] OR

"cost-utility"[tiab] OR "cost-benefit"[tiab] OR "economic evaluation"[tiab] OR

QALY[tiab] OR "quality-adjusted"[tiab])

## **3. EMBASE (Ovid), 1974 to July 2026 (Emtree)**

1. decompressive craniectomy/

2. (decompressive adj2 craniectom*).tw,kw.

3. (bifrontal craniectom* or temporal craniectom* or hemicraniectom*).tw,kw.

4. 1 or 2 or 3

5. traumatic brain injury/ or head injury/

6. (traumatic brain injur* or head injur* or head trauma or cerebral trauma or TBI).tw,kw.

7. 5 or 6

8. "cost effectiveness analysis"/ or "cost utility analysis"/ or "cost benefit analysis"/ or health economics/

9. (cost* or cost-effective* or cost-utility or cost-benefit or economic evaluation or QALY or quality-adjusted).tw,kw.

10. 8 or 9

11. 4 and 7 and 10

## **4. Cochrane Library (CENTRAL) and Cochrane Injuries Group Specialised Register, to July 2026**

```
#1  [mh "Decompressive Craniectomy"]
#2  (decompressive NEAR/2 craniectom*):ti,ab,kw
#3  (bifrontal craniectom* or temporal craniectom* or hemicraniectom*):ti,ab,kw
#4  #1 OR #2 OR #3
#5  [mh "Brain Injuries, Traumatic"] OR [mh "Craniocerebral Trauma"]
#6  ("traumatic brain injury" or "head injury" or "head trauma" or "cerebral trauma" or TBI):ti,ab,kw
#7  #5 OR #6
#8  [mh "Costs and Cost Analysis"]
#9  (cost* or "cost-effectiveness" or "cost-utility" or "cost-benefit" or "economic evaluation" or QALY or "quality-adjusted"):ti,ab,kw
#10 #8 OR #9
#11 #4 AND #7 AND #10
```

## **5. LILACS (via the Virtual Health Library / BVS), to July 2026**

(mh:"Decompressive Craniectomy" OR "decompressive craniectomy" OR

"craniectomia descompressiva" OR "craniectomia descompresiva" OR hemicraniectom*)

AND (mh:"Brain Injuries, Traumatic" OR "traumatic brain injury" OR

"traumatismo cranioencefalico" OR "head injury" OR TBI)

AND (mh:"Costs and Cost Analysis" OR cost* OR custo* OR costo* OR

"cost-effectiveness" OR "cost-utility" OR QALY)

*Bilingual (Portuguese, Spanish, and English) free-text terms were used for LILACS. Diacritics were omitted to maximize retrieval.*

Records retrieved from all sources were exported to a reference manager, deduplicated, and screened against the eligibility criteria by two reviewers independently, with disagreements resolved by consensus. Reasons for full-text exclusion are shown in the study-selection flow diagram (Figure 1).
